# Polypeptide Multilayer Self-Assembly Studied by Ellipsometry

**DOI:** 10.1155/2014/424697

**Published:** 2014-02-10

**Authors:** Marina Craig, Krister Holmberg, Eric Le Ru, Pablo Etchegoin

**Affiliations:** ^1^Department of Chemical and Biological Engineering, Chalmers University of Technology, Sven Hultins Gata 12, 412 96 Gothenburg, Sweden; ^2^Mölnlycke Health Care, P.O. Box 130 80, 402 52 Gothenburg, Sweden; ^3^School of Physical and Chemical Sciences, Victoria University of Wellington, P.O. Box 600, Wellington 6140, New Zealand

## Abstract

A polypeptide nanofilm made by layer-by-layer (LbL) self-assembly was built on a surface that mimics nonwoven, a material commonly used in wound dressings. Poly-L-lysine (PLL) and poly-L-glutamic acid (PLGA) are the building blocks of the nanofilm, which is intended as an enzymatically degradable lid for release of bactericides to chronic wounds. Chronic wounds often carry infection originating from bacteria such as *Staphylococcus aureus* and a release system triggered by the degree of infection is of interest. The dry nanofilm was studied with ellipsometry. The thickness of the nanofilm was 60% less in its dry state than in its wet state. The measurements showed that a primer was not necessary to build a stable nanofilm, which is practically important in our case because a nondegradable primer is highly unwanted in a wound care dressing. Added V8 (glutamyl endopeptidase) enzymes only showed adsorption on the nanofilm at room temperature, indicating that the PLL/PLGA “lid” may remain intact until the dressing has been filled with wound exudate at the elevated temperature typical of that of the wound.

## 1. Introduction

Self-assembly techniques have become increasingly popular as a tool to create controlled release systems for drug delivery. One such technique is layer-by-layer (LbL) self-assembly, which was pioneered by Decher [1, 2]. Based on this method targeted release of bactericides into an infected chronic wound can be developed. Overexposure of drugs often causes unwanted side effects, which may be avoided by regulating the drug release. Within chronic wound care, the primary function of a dressing is to absorb and retain wound exudate. However, a secondary function in the form of drug release may also play a crucial role for chronic wound healing. Many chronic wounds carry a bacterial infection [3, 4] generating high amounts of bacterial proteases [5] in the wound, which often disturbs the natural healing cascade. Such an infection is normally treated with oral antibiotics or with gels, creams, or wound dressings containing antimicrobial substances [6]. These are often applied in excess and may generate resistant bacteria [7, 8]. Unnecessary use of bactericides may be avoided by using an “intelligent dressing,” consisting of an absorbing nonwoven dressing material with a biodegradable polypeptide lid [9]. Antimicrobial agents are incorporated into the product in such a way that they are released into the infected wound when the wound exudate penetrates the material and the lid is degraded. The lid is made up of a thin polypeptide film and the degradation is caused by bacterial proteases from the wound, for example, from *Staphylococcus aureus*. The release of active substances will hence be triggered by the infection. The thin film is assembled at pH 7.4 and consists of two oppositely charged polypeptides deposited as alternating layers. The polypeptides of choice, cationic poly-L-lysine (PLL) and anionic poly-L-glutamic acid (PLGA), are both biocompatible and highly biodegradable [10, 11]. The procedure is an example of the LbL technique. Quartz crystal microbalance with dissipation monitoring (QCM-D) has been used to study both the build-up and the degradation of the polypeptide film [9]. The underlying substrate was a model surface tailored by the use of self-assembling alkane thiols to give the same wetting characteristics as an existing nonwoven material (regenerated cellulose) used in wound dressings. However, the QCM-D method gives quantitative information about the wet film and the values obtained for adsorption and desorption at the underlying surface reflect the total amount of material on the surface, that is, the two polypeptides as well as water incorporated into the polypeptide film. Since we are interested in obtaining quantitative information about the polypeptide layers *per se*, an alternative measurement technique is needed and ellipsometry, which is an optical method that records adsorption and desorption of organic molecules at a surface and disregards the water that comes along with the adsorbate, was chosen as a suitable method for the purpose.

## 2. Experimental and Methods

### 2.1. Preparation of Tailored Gold Surfaces

Clean (acid etching) and ready to use 41 nm gold sensors (XanTec bioanalytics, Düsseldorf, Germany) were tailored to imitate a regenerated cellulose or nonwoven surface using a 2.0 mM mixture of 60 mol% 11-hydroxy-1-undecanethiol and 40 mol% 1-undecanethiol (Sigma Aldrich) in 99.5% ethanol. The sensors were immersed in the alkane thiol solution for 16–20 hours before rinsing in 99.5% ethanol and drying with nitrogen gas before use. Gold substrates used for a second or third time were cleaned in a Plasma Etch situated in a cleanroom. Chosen effect, gas, time/cycle, and amount of cycles were 100 W, O_2_, 1 min 10 sec/cycle, and two cycles. Contact angle measurements of the substrates were used as a control of the cleanliness.

### 2.2. Preparation of PLL and PLGA Solutions and Multilayer Build-Up

Poly-L-lysine (PLL) (MW = 30–70 kDa) and poly-L-glutamic acid (PLGA) (MW = 50–100 kDa) were purchased from Sigma Aldrich. A 0.1 mM buffer solution was prepared from Tris-HCl (Sigma Aldrich) and ultrapure water (Milli-Q resistivity > 18 MΩ cm). NaCl (Fluka) (0.1 M) was added to the 0.1 mM Tris-HCl solution followed by addition of either PLL or PLGA in an amount corresponding to 1 mg/mL. Both polyelectrolyte solutions were placed on a magnetic stirrer for 1 hr before use. All end solutions had a pH of 7.4. The SAM covered gold surfaces were used for layer-by-layer (LbL) assembly of PLL/PLGA multilayers. Three PLL/PLGA bilayers were deposited by immersion into each polypeptide solution, respectively, with rinsing steps using Tris-HCl buffer after each immersion. The first PLL layer was immersed in solution for 30 mins allowing a stable cationic foundation for the rest of the build-up, whereafter the deposition and rinsing were performed with 10 min intervals. All (PLL/PLGA)_3_ samples were dried in N_2_ gas and placed for ellipsometry measurements in air and ambient temperature. Also, all samples were stored in air and remeasured after 24 hours to make sure that the samples were as dry as possible.

### 2.3. LbL and PLL/PLGA Nanofilms

Self-assembly with the LbL technique can be described by creating an overcompensated surface charge by depositing one charged polymer on a surface which in turn enables further deposition of an oppositely charged polymer and so on [12]. Films consisting of three PLL/PLGA bilayers were formed in this study. Both PLL and PLGA are weak polyelectrolytes and therefore create dynamic layers, a process that needs strict control of several parameters, for example, polyelectrolyte concentration, ionic strength, and so forth [13, 14].

### 2.4. Preparation of Enzyme Solutions

Endoproteinase Glu-C from *Staphylococcus aureus* V8 (type XVII-B) and the reference enzyme, trypsin from bovine pancreas (type I, ~10,000 BAEE units/mg protein), were both purchased from Sigma Aldrich. The preparations of enzyme solutions included 1 : 100 [9, 15] dilution with ultrapure water (MilliQ, resistivity > 18 MΩ cm) and were used within 15 mins to ensure highest possible enzyme activity. Samples with three PLL/PLGA bilayers were immersed into the enzyme solutions, respectively, for possible degradation of peptides.

### 2.5. Ellipsometry

Ellipsometry was performed at ambient temperature on an instrument from Beaglehole Instruments (New Zealand). More information about the measuring procedure can be found in the Supporting Information (see Supplementary Material available online at http://dx.doi.org/10.1155/2014/424697).

## 3. Results and Discussion

### 3.1. Ellipsometry Study and Data Analysis of the Build-Up of the PLL/PLGA Multilayer

Three PLL/PLGA bilayers were assembled directly on the alkane thiol model surface without the use of a primer. A primer such as poly(ethylene imine) is often used for LbL assemblies of polypeptides [16] but for this specific application it is vital that all components are susceptible to peptidase-catalyzed degradation and also are biocompatible. Thus, we chose not to involve a primer. The clean gold surface was initially studied in the ellipsometer. Using the TFCompanion software and a double-layer model with the Marquardt-Levenberg algorithm, a thickness of 406 ± 6.7 Å was obtained, which agreed well with the manufacturer's specification of a thickness of 410 Å. At 633 nm the *n* for gold was 0.182 and *k* was 3.436. It is known that cleaning the gold by plasma treatment can cause an increase of the surface roughness. The gold substrates were therefore always remeasured after cleaning and fitted to the two-layer model using a Bruggeman effective medium approximation (EMA) layer on top of the bulk layer. However, the surface roughness was estimated to be very small even after plasma treatment, with a thickness of 371 ± 6.9 Å. Care was taken that the values were repeatable throughout each test session. The next step was to perform ellipsometry measurements on the SAM coated surface. It was not considered appropriate to add an EMA layer on top of a thin monolayer, which is why a two-layer model for SAM on gold was used with *n* assumed to be 1.4999 at 633 nm [17]. The error of using an *n* value of 1.5 ± 0.05 is estimated to be less than 1 Å [18]. Again, the surface roughness was very small. The SAM thickness was 18 ± 2 Å, which was appropriate since the theoretical value is 17 Å [19]. This surface is then the starting point for the LbL assembly of the two polypeptides, the cationic PLL and the anionic PLGA. These were assembled in three bilayers, always starting with deposition of PLL on the SAM surface. Thus, the entire composition on which the ellipsometry measurements were conducted can be written (Au-SAM)-(PLL/PLGA)_3_, where the deposited polypeptide film thickness was solved by using a four-layer model using polypeptide bulk and polypeptide EMA layers on top. Considering the low amount of bilayers assembled, the refractive index was assumed to be rather low (*n* = 1.4) and *k* = 0 at 633 nm for both polypeptides [20]. In literature it has been described that the refractive index increases with increasing bilayers, as is the case for the PLL/PLGA film.

**Figure 1:**
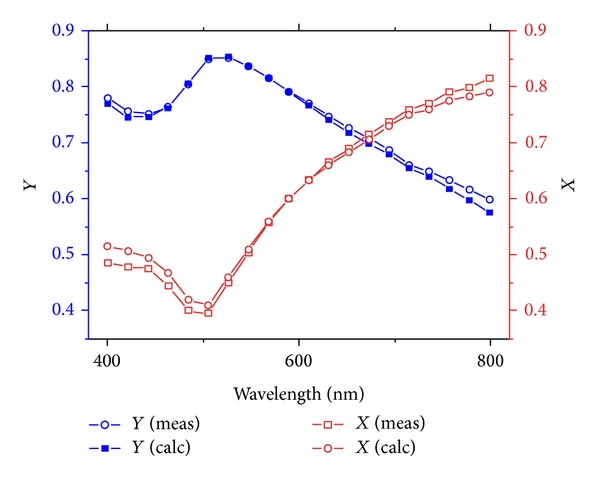
Measured data for *X* (related to the real part) and *Y* (related to the imaginary part) fitted well with the calculated data using TFCompanion. The filmstack consists of (Au-SAM)-(PLL/PLGA)_3_, including an air content of 10% in the polypeptide layers. The polypeptide film has *n* = 1.4 at 633 nm. The calculation is performed using the Marquardt-Levenberg and a three-layer model. Ellipsometry equations for *X* and *Y* can be found in Supporting Information.

The ellipsometry raw data was exported to the TFCompanion software and models were created using raw data originating from the gold substrates modified by alkane thiols as the starting point. Both polypeptides are weak polyelectrolytes and LbL films from such polymers have been reported not to be rigid [20]. This was also seen when fitting the raw data to the model. Air was added in an amount of about 10% of the volume of the polypeptides as one film component, which resulted in a MSE (mean squared error) of 0.011 for all measurements. The final fit of the model of choice for the Au-SAM-(PLL/PLGA)_3_ film can be seen in Figure 1. Three PLL/PLGA bilayers deposited on the alkane thiol-covered gold surface gave a dry thickness of approximately 40 ± 3.4 Å. A QCM-D study gave a thickness of the wet (PLL/PLGA)_3_ film of 100 ± 10 Å [9]. Hence one may conclude that about 60% of the wet layer consisted of water. This is reasonable and agrees with previously reported values in the literature. It has been claimed that the large amount of bound water in the film is due to the polypeptides adsorbing in loops and tails, which favors storage of water in the film [20]. It is interesting that roughly the same dry content of the film is obtained in this work, in which no primer was used in the LbL process, as in earlier work with a primer present. Thus, the primer seems not to be needed on the alkane thiol surface used in the present systems, which, as discussed above, is a considerable advantage from an application point of view.

### 3.2. Ellipsometry Study and Data Analysis of the Enzymatic Degradation of the PLL/PLGA Multilayers

**Figure 2:**
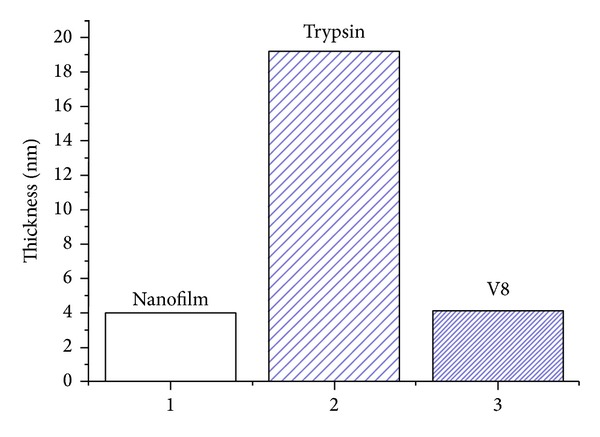
The dry nanofilm thickness of 40 ± 3.4 Å nm increased considerably when bovine trypsin adsorbed to the film surface, whereas it remained less affected when exposed to the V8 enzyme, at ambient temperature.

After the ellipsometry measurement the (Au-SAM)-(PLL/PLGA)_3_ substrate was immersed in buffer solution and allowed to swell for at least 30 minutes before use. The samples were then moved to a new container and a solution of either trypsin (bovine) or V8 glutamyl endopeptidase (*Staphylococcus aureus*) was added. Two different degradation times were chosen for each enzyme solution, 3 hours and 16 hours. The enzymes were either added at one time or up to three times during the degradation period. The samples were then rinsed with Milli-Q water and dried in nitrogen gas before ellipsometry measurements were performed. The assumed *n* value was 1.48 at 633 nm for both enzymes [21] (*k* = 0), which is an appropriate value for proteins [22, 23]. As can be seen from Figure 2, trypsin adsorbed readily to the polypeptide surface and increased the thickness up to 5 times. This indicates that the overall positively charged trypsin (pH 7.4) adsorbs readily on top of the thin polypeptide film (negatively charged surface) but the adsorption evidently does not result in any visible enzymatic degradation. This is similar to what was seen in a QCM-D study of the same system [9]. Despite a large increase in thickness it was difficult to rule out any catalytic activity; however, as trypsin prefers to catalyze positively charged substrates, the polypeptide film ending with negatively charged PLGA would not be ideal for catalysis. When modeling the adsorbed trypsin, a five-layer model was used; however, only the thicknesses of the trypsin and trypsin_EMA layers were variable. The roughness of the film was very low ending up with a trypsin thickness of 65 ± 3.7 Å (MSE = 0.013), that is, a total polypeptide/enzyme thickness of 105 ± 3.7 Å.

The bacterial protease V8 behaved differently. The thickness of the nanofilm did not change much on exposure to the solution of V8 enzyme, as can also be seen from Figure 2. A four-layer model was used for the V8 enzyme's adsorption to the film, since the layer on top of the polypeptide film was very thin. The best fit received a polypeptide/enzyme thickness of 42 ± 2.9 Å. The immediate interpretation of this result is that the V8 enzyme did not interact with the polypeptide film, as both enzyme and the polypeptide surfaces were negatively charged. However, Craig et al. showed that this enzyme catalyzed degradation of the LbL film provided that it was terminated by the anionic PLGA. The reason for this is that the V8 peptidase is known to be reactive in catalyzing cleavage of Glu-X bonds, that is, peptide bonds involving a glutamate residue [24, 25]. The LbL film studied in this work had PLGA as the terminating layer. However, the temperatures differed. Whereas the QCM-D measurements were performed at 32°C, which was intended to mimic the temperature of a typical wound, the ellipsometry experiments were performed at ambient temperature. Thus, the temperature is vital and no or little enzymatic degradation occurred at room temperature. This is a practically important piece of information because it indicates that the wound dressing with the antimicrobial agents covered by the polypeptide lid remains intact until it is contacted by the exudate from a chronic wound at the approximate skin temperature of 32°C. However, the V8 protease may not be entirely inactive also at ambient temperature. The surface that has been exposed to the V8 peptidase solution seems to be slightly rougher immediately after the treatment (±6 Å) than after one or two days, when all measuring points ended up at the same value (±1 Å). This induced roughness of the surface may indicate enzymatic cleavage of the top layer of the film, that is, predominantly of PLGA.

## 4. Conclusion

The (PLL/PLGA)_3_ nanofilm was measured with ellipsometry to study the thickness in its dry state. When comparing with the film's wet and dry thicknesses, it is clear that about 60% of the wet film consists of water. This result is in accordance with previously reported values from similar systems despite the fact that in the present investigation the polypeptides were adsorbed directly to a tailored gold surface imitating nonwoven and not to a surface treated with a primer such as PEI, which is the normal procedure. This indicates that the character of the film without primer is similar to that with primer. This is practically important in our case because a nondegradable primer is highly unwanted in a wound care dressing. The enzymatic degradation of the nanofilm was also monitored by ellipsometry. Bovine trypsin adsorbed at the polypeptide surface but there were no indications of an enzymatic degradation of the LbL film even after sequential addition of the peptidase. Also the V8 glutamyl endopeptidase from *Staphylococcus aureus* seemed not to cause much degradation of the polypeptide nanofilm. However, the present ellipsometry study was distinguished by being conducted at ambient temperature, whilst a previous study was performed at 32°C [9], a temperature chosen to mimic the temperature of the wound. This difference with respect to temperature dependency indicates that the PLL/PLGA “lid” may remain intact until the dressing has been filled with wound exudate with the elevated temperature typical of that of the wound.

## Supplementary Material

The supplementary information for the research article “Polypeptide Multilayer Self-Assembly Studied by Elliposmetry” contains information about the ellipsometer and the ellipsometric parameters used in this study, as well as information about the main equations in ellipsometry.

## References

[1] Decher G, Hong JD, Schmitt J (1992). Buildup of ultrathin multilayer films by a self-assembly process: III. Consecutively alternating adsorption of anionic and cationic polyelectrolytes on charged surfaces. *Thin Solid Films*.

[2] Decher G (1997). Fuzzy nanoassemblies: toward layered polymeric multicomposites. *Science*.

[3] Bowler P (1998). The anaerobic and aerobic microbiology of wounds: a review. *Wounds*.

[4] Landis SJ (2008). Chronic wound infection and antimicrobial use. *Advances in Skin & Wound Care*.

[5] Greener B, Hughes AA, Bannister NP, Douglass J (2005). Proteases and pH in chronic wounds. *Journal of Wound Care*.

[6] Burell RE (2003). A scientific perspective on the use of topical silver preparations. *Ostomy Wound Manage*.

[7] Percival SL, Woods E, Nutekpor M, Bowler P, Radford A, Cochrane C (2008). Prevalence of silver resistance in bacteria isolated from diabetic foot ulcers and efficacy of silver-containing wound dressings. *Ostomy Wound Management*.

[8] Flattau A, Lowy FD (2008). Antibiotic-resistant gram-negative bacteria in deep tissue cultures. *International Wound Journal*.

[9] Craig M, Bordes R, Holmberg K (2012). Polypeptide multilayer self-assembly and enzymatic degradation on tailored gold surfaces studied by QCM-D. *Soft Matter*.

[10] Richert L, Arntz Y, Schaaf P, Voegel J-C, Picart C (2004). ph dependent growth of poly(l-lysine)/poly(l-glutamic) acid multilayer films and their cell adhesion properties. *Surface Science*.

[11] Jessel N, Atalar F, Lavalle P (2003). Bioactive coatings based on a polyelectrolyte multilayer architecture functionalized by embedded proteins. *Advanced Materials*.

[12] Kickelbick G (2007). *Hybrid Materials: Synthesis, Characterization, and Applications*.

[13] Decher G, Schlenhoff JB (2003). *Multilayer Thin Films: Sequential Assembly of Nanocomposite Materials*.

[14] Gergely C, Bahi S, Szalontai B (2004). Human serum albumin self-assembly on weak polyelectrolyte multilayer films structurally modified by pH changes. *Langmuir*.

[15] Schmidtchen A

[16] Halthur TJ, Claesson PM, Elofsson UM (2004). Stability of polypeptide multilayers as studied by in situ ellipsometry: effects of drying and post-buildup changes in temperature and pH. *Journal of the American Chemical Society*.

[17] Bain CD, Troughton EB, Tao Y-T, Evall J, Whitesides GM, Nuzzo RG (1989). Formation of monolayer films by the spontaneous assembly of organic thiols from solution onto gold. *Journal of the American Chemical Society*.

[18] Wasserman SR, Whitesides GM, Tidswell IM, Ocko BM, Pershan PS, Axe JD (1989). The structure of self-assembled monolayers of alkylsiloxanes on silicon: a comparison of results from ellipsometry and low-angle X-ray reflectivity. *Journal of the American Chemical Society*.

[19] Heise A, Menzel H, Yim H (1997). Grafting of polypeptides on solid substrates by initiation of N-carboxyanhydride polymerization by amino-terminated self-assembled monolayers. *Langmuir*.

[20] Halthur TJ, Elofsson UM (2004). Multilayers of charged polypeptides as studied by in situ ellipsometry and quartz crystal microbalance with dissipation. *Langmuir*.

[21] Voros J (2004). The density and refractive index of adsorbing protein layers. *Biophysical Journal*.

[22] Hand DB (1935). The refractivity of protein solutions. *The Journal of Biological Chemistry*.

[23] Cuypers PA, Hermens WT, Hemker HC (1977). Ellipsometric study of protein film on chromium. *Annals of the New York Academy of Sciences*.

[24] Breddam K, Meldal M (1992). Substrate preferences of glutamic-acid-specific endopeptidases assessed by synthetic peptide substrates based on intramolecular fluorescence quenching. *European Journal of Biochemistry*.

[25] Sorensen SB, Sorensen TL, Breddam K (1991). Fragmentation of protein by S. aureus strain V8 protease. Ammonium bicarbonate strongly inhibits the enzyme but does not improve the selectivity for glutamic acid. *FEBS Letters*.

