# Supplementary material for: Polypeptide Multilayer Self-Assembly Studied by Ellipsometry
Source: J Drug Deliv. 2014 Feb 10;2014:424697. doi: 10.1155/2014/424697 (PMC3934801; doi:10.1155/2014/424697)
Supplement: Supplementary file 1 — The supplementary information for the research article “Polypeptide Multilayer Self-Assembly Studied by Elliposmetry” contains information about the ellipsometer and the ellipsometric parameters used in this study, as well as information about the main equations in ellipsometry. [file 424697.f1.pdf]

Supporting Information

Ellipsometry

Ellipsometry is an optical technique used for studies of the dielectric properties of thin films. The technique has been thoroughly described in the literature [1, 2]. In brief, the change of polarization of light reflected (or transmitted) off a sample gives information about the thin film. Using advanced software this information is available for interpretation and modeling of data, *e.g.* calculation of film thickness. In this study an ellipsometer from Beaglehole Instruments (New Zealand) for wavelengths between 196-2000 nm was used. The ellipsometer operates with an elasto-optic modulator. TFCompanion software (Semiconsoft, Massachusetts, US) was used for the data analysis. All measurements were performed at ambient temperature.

Initial measurements were performed in UV light (200-410 nm) using a deuterium lamp and in visible light (400-799 nm) using a halogen lamp, respectively, to avoid possible disturbances from the reflective gold surface. Data analysis showed a good fit between 400-799 nm with the monochromator locked at 633 nm. The measurements in UV light did not give a good fit for the current system, however. Hence, all the data recorded in this study were obtained from measurements in visible light with an angle of incidence (AOI) of 60°. All samples were measured once, then rotated 90° and later 180° for additional measurements to study possible sample anisotropy. Additional measurements were performed when the gold surfaces had been plasma treated.

The main equations in ellipsometry are presented below:

$$r = \frac{r_p}{r_s} = Re(r) + i \times Im(r)$$
$$X = \frac{2}{1+\rho^2} Re(r)$$
$$Y = \frac{2}{1+\rho^2} Im(r)$$
$$\rho = |r|$$

r_p and r_s denote the complex amplitude reflectivities (i.e. Fresnel reflection coefficients) for electric field components polarized parallel and perpendicular to the plane of incidence, respectively. The ratio *r* is in general complex, and our ellipsometric measurements allow us to measure both its real and imaginary part, or equivalently the quantities *X* and *Y* defined above, on which the analysis/modeling is carried out using standard Fresnel equations for the multilayer interface, ρ is real and denotes the complex modulus of *r*.

References

[1] Ed. by Tompkins H. G.; Irene E. A. Handbook of Ellipsometry, 2005.
[2] Azzam R. M. A.; Bashara N. M. Ellipsometry and polarized light, 1987.
